# The Applicability of Oxidative Stress Biomarkers in Assessing Chromium Induced Toxicity in the Fish *Labeo rohita*

**DOI:** 10.1155/2014/782493

**Published:** 2014-09-14

**Authors:** Kanchan Kumari, Ankur Khare, Swati Dange

**Affiliations:** EIRA Division, CSIR-National Environmental Engineering Research Institute (NEERI), Nehru Marg, Nagpur 440020, India

## Abstract

The evaluation of metal's toxicity in freshwater is one of the imperative areas of research and there is an emergent concern on the development of techniques for detecting toxic effects in aquatic animals. Oxidative stress biomarkers are very useful in assessing the health of aquatic life and more in depth studies are necessary to establish an exact cause effect relationship. Therefore, to study the effectiveness of this approach, a laboratory study was conducted in the fish *Labeo rohita* as a function of hexavalent chromium and the toxicity indices using a battery of oxidative stress biomarkers such as catalase (CAT), superoxide dismutase (SOD), and glutathione reductase (GR) in the liver, muscle, gills, and brain have been studied along with biometric parameters, behavioral changes, and Cr bioaccumulation. A significant increased HSI was observed in contrast to CF which reduced significantly. SOD, CAT, and GR activity increased significantly in all the tissues of treated fishes. The bioaccumulation of Cr was highest in liver followed by gills, muscle, and brain. This study highlights the significance of using a set of integrated biomarker and advocate to include these parameters in National Water Quality Monitoring Program in areas potentially polluted with metals to assess the health of the ecosystem.

## 1. Introduction

Heavy metals play a crucial role in various biological functioning of aquatic organisms and remain present in trace amount in the body, that is, not exceeding 1 *μ*g/g. However only slight increase in the concentration leads to high level of toxicity in different organs. Aluminium, arsenic, chromium, cobalt, copper, iron, manganese, molybdenum, nickel, selenium, tin, vanadium, and zinc are essential heavy metals for one or more organisms. But industrial effluents containing toxic and hazardous substances, including heavy metals, consequently lead to the pollution of aquatic ecosystem [1]. Heavy metals are competent of inducing toxicity in living organisms because of their capability of interacting with the nuclear proteins and nucleic acids causing oxidative deterioration of biomolecules [2]. The unifying factor determining the toxicity and carcinogenicity for heavy metals is the generation of reactive oxygen and nitrogen species [3] resulting in cellular damage like enzymes depletion and DNA damage [4]. Due to the fact that even trace amount of some heavy metals can exhibit high toxicity to aquatic biota and human, there is an increasing interest in studying interaction of these metals in the aquatic environments.

Aquatic system is an ultimate sink of heavy metal pollutants and since aquatic animals tend to accumulate heavy metals from various sources including sediments, soil erosion and runoff, air depositions of dust and aerosol, and discharges of waste water [5, 6], they provide the insights of toxicity mechanisms induced by these heavy metals. Among these heavy metals, cadmium, lead, copper, and chromium pose major problems to aquatic life. Chromium (Cr) is one of the most common contaminants found in surface and ground water. Welding, grinding, and polishing of stainless steel are the principal sources of chromium pollution, while the other sources include burning of fossil fuels and waste incineration [7]. Chromium compounds are widely used as mordant and dyes in textile industry, chrome electroplating, anodizing, and dipping and it is used as oxidants and catalysts in the manufacture of products such as saccharin, in bleaching and purification of oils, fats, and chemicals and as agents to increase the antiwetting by water insolubility of various products such as glues, inks, and gels. Because of the wide industrial uses of Cr metal and its compounds, anthropogenic activities have become the most significant contributor to environmental contamination.

The hexavalent chromium when present in excess amount induces toxic effects in the cells [8] like genotoxicity [9, 10] and oxidative damage [11–13]. Oxidative damage primarily occurs through production of reactive oxygen species (ROS) and can damage lipids, proteins, and DNA contributing to loss of activity and structural integrity of enzymes and may activate inflammatory processes [14]. In most cases, the abnormal generation of ROS, which can result in significant damage to cell structure, is considered as an important signal of oxidative damage [15]. Oxidative stress is induced as a result of the three factors: (a) an increase in oxidant generation, (b) a decrease in antioxidant protection, and (c) failure to repair oxidative damage [16]. Superoxide (O^−2^), one of the parental forms of intracellular ROS, is a very reactive molecule, but it can be converted to H_2_O_2_ by superoxide dismutase (SOD) and then to oxygen and water by several enzymes including catalase (CAT) and glutathione reductase (GR). Therefore, examining the change in activity of antioxidant enzymes such as SOD, CAT, and GR shall be an effective method of denoting oxidative stress and changes in their activity and other biomarkers could be the possible tools in aquatic toxicological research.

The evaluation of toxic effects of metals in terrestrial and aquatic ecosystems is one of the imperative areas of recent research and there is an emergent concern on the development of technique for detection of toxic effects in aquatic animals [17]. Fishes are an important source of human diet and numerous studies have been carried out on metal pollution in different edible fish species [18, 19]. Industrial effluents, agricultural runoffs, transport, burning of fossil fuels, and domestic wastes append to the heavy metals in the water bodies owing to their easy uptake into the food chain and bioaccumulation processes. The toxic effects of heavy metals have been examined, including bioaccumulation [20] and the instance of metal accumulation in fish tissues can be utilized as effective indicators of environmental contamination [21]. Many authors have advocated to use the oxidative biomarkers in assessing the health of aquatic life [22, 23] and more in depth studies are necessary to establish an exact cause effect relationship. Therefore, to study the effectiveness of this approach, a laboratory study was conducted in the fish* Labeo rohita* as a function of heavy metal chromium. The rationale of this research is to study the chromium induced oxidative stress along with some biometric assays and to quantify the accumulation of chromium in different tissues of* Labeo rohita*, a most common edible carp fish, and correlate the concentration of metals with respect to their toxic effects on various fish species. The acquired information would further help in the formulation of strategies for treating chromium polluted water bodies and making the river water safe for survival of aquatic life.

## 2. Materials and Methods

### 2.1. Experimental Fishes

*Labeo rohita*, a common carp, weight 59 g ± 8.2; length 16 cm ± 1.46, were obtained from the local hatchery maintained by College of Fishery Science, Telankhedi, Nagpur, and acclimatized according to the method of Kumari and Sinha [24]. The water quality parameters measured included pH 7.8 ± 0.5, temperature 24.0 ± 1.14°C, dissolved oxygen 7.25 mgL^−1^, total alkalinity 90 mgL^−1^, and total hardness 40.52 ± 3.2 mgL^−1^ as CaCO_3_. Photoperiod was 12 : 12 light-dark cycle. Fishes were fed commercial fish food and acclimatized for 14 days prior to the beginning of the experiment.

### 2.2. Experimental Design

Following acclimatization, short term test of acute toxicity over a period of 96 hrs was performed on the fishes following the renewal of bioassay. LC_50_ values were determined by EPA Probit Analysis Program [25]. Fishes were exposed with 48.3 ppm (1/3rd of LC_50_ of potassium dichromate). The behavior and condition of the fishes were noted every 24 hr up to 96 hrs. Control fishes were also held under acclimatized conditions and monitored concurrently and no mortality was observed in this group. After 24, 48, 72, and 96 hrs and 15 days of exposure, fishes from treatment and control group were sacrificed for different assays. Collection of blood was done according to the method of Kumari and Sinha [26]. Tissues were quickly removed in the following order: gills, brain, liver, and muscle and processed immediately for different assays. For spectrophotometric analysis of different assays, UV Spectrophotometer (Shimadzu; Model- UV-1800) equipped with temperature control system was used.

### 2.3. Biometrics Assay

Two biometric parameters were calculated, the condition factor (CF) and the hepatosomatic index (HSI). The condition factor of each fish was calculated using the method of Salam and Davies [27] and the hepatosomatic index was calculated using the method of Kumari [28].

### 2.4. Assay of Total Protein

To calculate the enzyme activity, total protein content was determined in different tissues under study based on Biuret method [29].

### 2.5. Assay of Catalase (CAT, EC 1.11.1.6)

Catalase was assayed according to the method of Sinha [30] with slight modifications. The reagent used includes phosphate buffer (0.1 M, pH 7.0) and 30% hydrogen peroxide solution.

Known amount of tissue was homogenized in phosphate buffer. The reaction mixture includes 0.1 mL of hydrogen peroxide, 1.95 mL phosphate buffer, and 0.05 mL tissue extract. Change in absorbance was recorded at 240 nm and the enzyme activity was expressed as mmol^−1^ H_2_O_2_ min^−1 ^mg^−1^ protein.

### 2.6. Assay of Superoxide Dismutase (SOD, EC 1.15.1.1)

SOD activity was determined according to the method of Beauchamp and Fridovich [31] with little modifications. The reagents used included phosphate buffer (0.1 M, pH 7.5), riboflavin (24 *μ*M), nitro blue tetrazolium (NBT) (840 *μ*M), Na_2_EDTA (1.2 mM), and methionine (150 mM).

The reaction mixture containing 1.95 mL phosphate buffer, 0.25 mL riboflavin, 0.25 mL methionine, 0.25 mL Na_2_EDTA, 0.25 mL NBT, and 0.05 mL tissue extract was pipetted in 4 glass tubes. Another set of 4 tubes was prepared adding 0.05 mL of phosphate buffer instead of enzyme extract. Three tubes from each set were then placed on shaker at 25°C in fluorescent light for 15 minutes and the last one was kept in dark at 25°C (reference sample, in darkness free radicals are not generated). After the incubation period the change in the absorbance was measured at 560 nm using respected dark-incubated sample as reference for test samples for each series. The SOD activity was expressed in terms of relative enzyme activity U/mg protein.

### 2.7. Assay of Glutathione Reductase (GSSG-R or GR, EC 1.6.4.2)

Glutathione reductase (GR; EC 1.6.4.2) activity was assayed as described by Carlberg and Mannervik [32], with some modifications, by measuring the oxidation of NADPH at 340 nm. The reaction mixture consisted of phosphate buffer (0.1 M, pH 7.5), 50 mM MgCl_2_, 16 M oxidized glutathione (GSSG), and 8 mM NADPH and was initiated by addition of supernatant. The activity of GR was calculated based on tissue protein concentration and expressed as relative enzyme activity.

### 2.8. Bioaccumulation of Chromium

Aquatic organisms have the ability to absorb and accumulate heavy metals that can pose a long term effect on the health of the organism and probably on the ecosystem. Bioaccumulation pattern of hexavalent chromium in different fish tissues was studied. The procedures adopted for heavy metal analysis in fish tissues are based on the method 3052 of EPA [33]. The bioaccumulation of chromium was studied after acute (short term exposure till 96 hrs) and chronic (long term exposure till 15 days) exposure in different tissues of* Labeo rohita* using ICP-OES.

### 2.9. Statistical Analysis

All statistical analyses were performed with SPSS statistical program. All experimental data were expressed as mean ± standard deviation (SD). Significant differences between experimental and control groups were compared by One-Way ANOVA (analysis of variance) followed by Least Significant Difference (LSD) (*P* < 0.05 and 0.001) using the Statistics Package (SPSS) program Version 7.

## 3. Results

### 3.1. Behavioral Changes in the Fishes under Study

While conducting LC_50_ assays some selective behavioral changes were also noticed as a function of hexavalent chromium and have been presented in Table 1.

### 3.2. Biometric Parameters

#### 3.2.1. Hepatosomatic Index (HSI)

**Figure 1:**
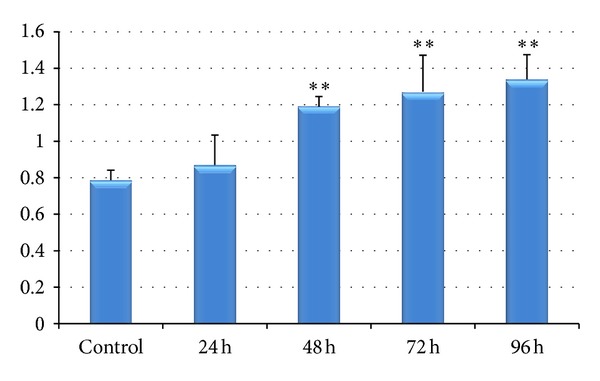
Effect of hexavalent chromium on HSI of* Labeo rohita* (values are mean ± S.D.); S.D. = standard deviation; ** = significant at (*P* < 0.05).

Figure 1 shows that chromium treated fishes exhibited a significantly increased hepatosomatic index (HSI) than that observed in control fishes. At the same time, treatment period was also observed to have a significant effect on the HSI as it increased progressively with the increasing period of exposure.

#### 3.2.2. Condition Factor (K)

**Figure 2:**
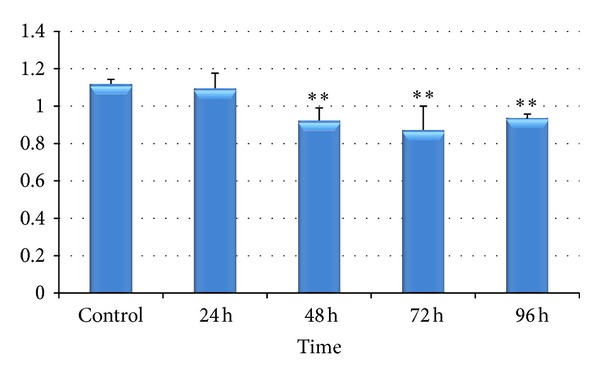
Effect of hexavalent chromium on CF of* Labeo rohita* (values are mean ± S.D.); S.D. = standard deviation; ** = significant at (*P* < 0.05).

Condition factor indicates changes in energy storage and metabolism due to environmental stressors. In this parameter, length and weight relationship are commonly used as indicators of robustness. Significant reduction was noticed in condition factor of chromium treated fishes (Figure 2).

### 3.3. Biomarkers of Oxidative Stress

#### 3.3.1. Catalase Activity

**Figure 3:**
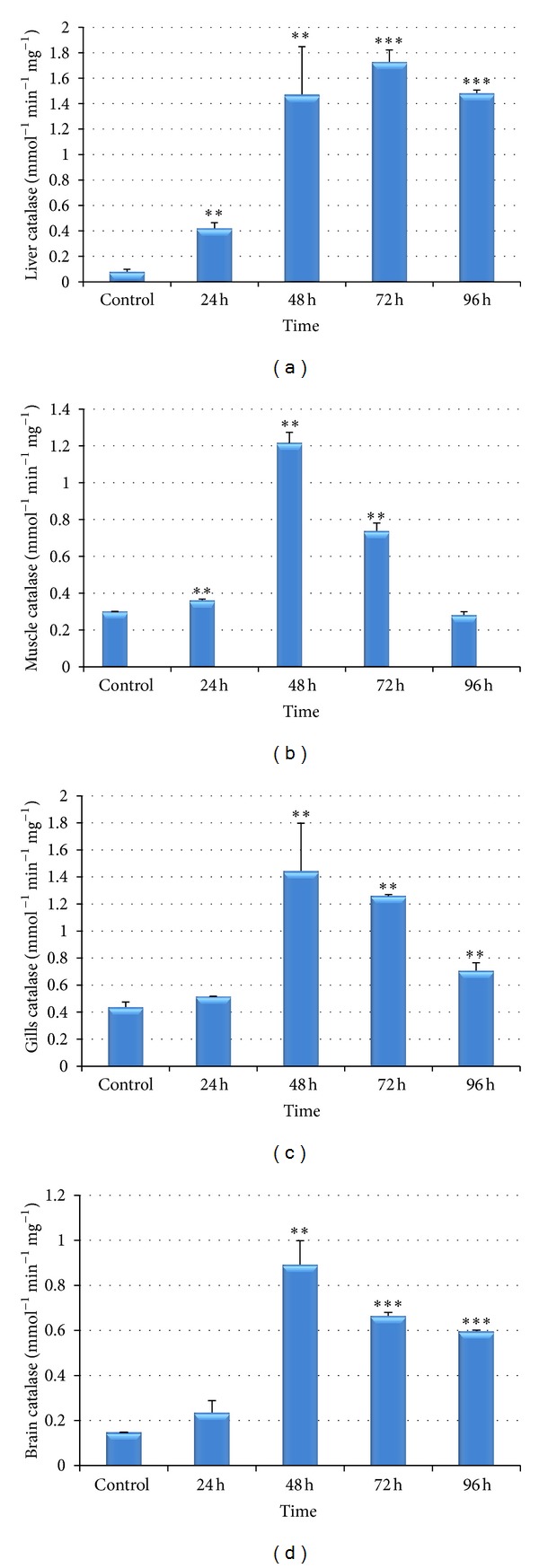
(a)–(d) Effect of hexavalent chromium on catalase activity in liver (a), muscle (b), gills (c), and brain (d) of* Labeo rohita* (values are mean ± S.D.); S.D. = standard deviation; ** = significant at (*P* < 0.05); *** = significant at (*P* < 0.001).

Figures 3(a)–3(d) show the effects of Cr(VI) on catalase activity in liver, muscle, gills, and brain of the fish* Labeo rohita*. A significant increase was observed in catalase activity in all the tissues under study in comparison to control fishes. The activity reached the maximum value in all the tissues during the period of 48 hrs except in liver tissue where it achieved its maximum level during 72 hrs.

#### 3.3.2. Superoxide Dismutase (SOD) Activity

**Figure 4:**
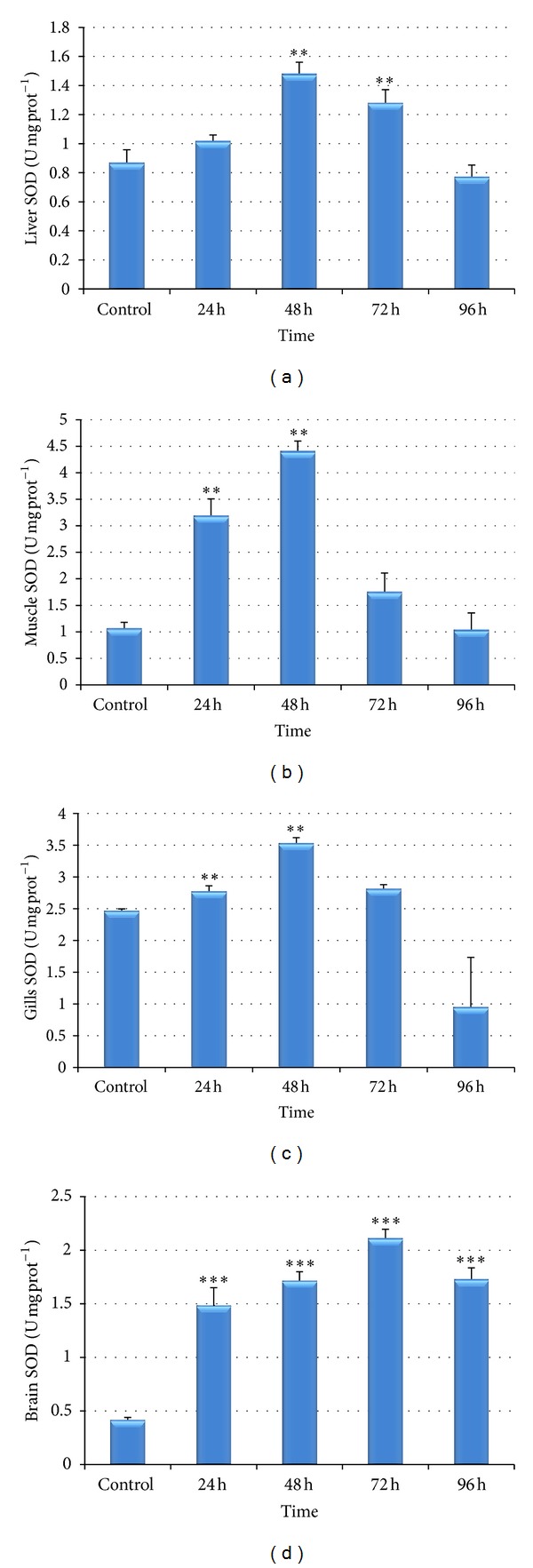
(a)–(d) Effect of hexavalent chromium on superoxide dismutase activity in liver (a), muscle (b), gills (c), and brain (d) of* Labeo rohita* (values are mean ± S.D.); S.D. = standard deviation; ** = significant at (*P* < 0.05); *** = significant at (*P* < 0.001).

To verify the presence of oxidative stress, SOD was also analyzed in liver, muscle, gills, and brain of the fish (Figures 4(a)–4(d)). Compared with the control, the activity of superoxide dismutase increased in chromium treated fishes during 24 to 72 hrs and decreased in 96 hrs in all the tissues except in brain where the activity of SOD was higher than normal even after 96 hrs of treatment period.

#### 3.3.3. Glutathione Reductase Activity (GSSG-R)

**Figure 5:**
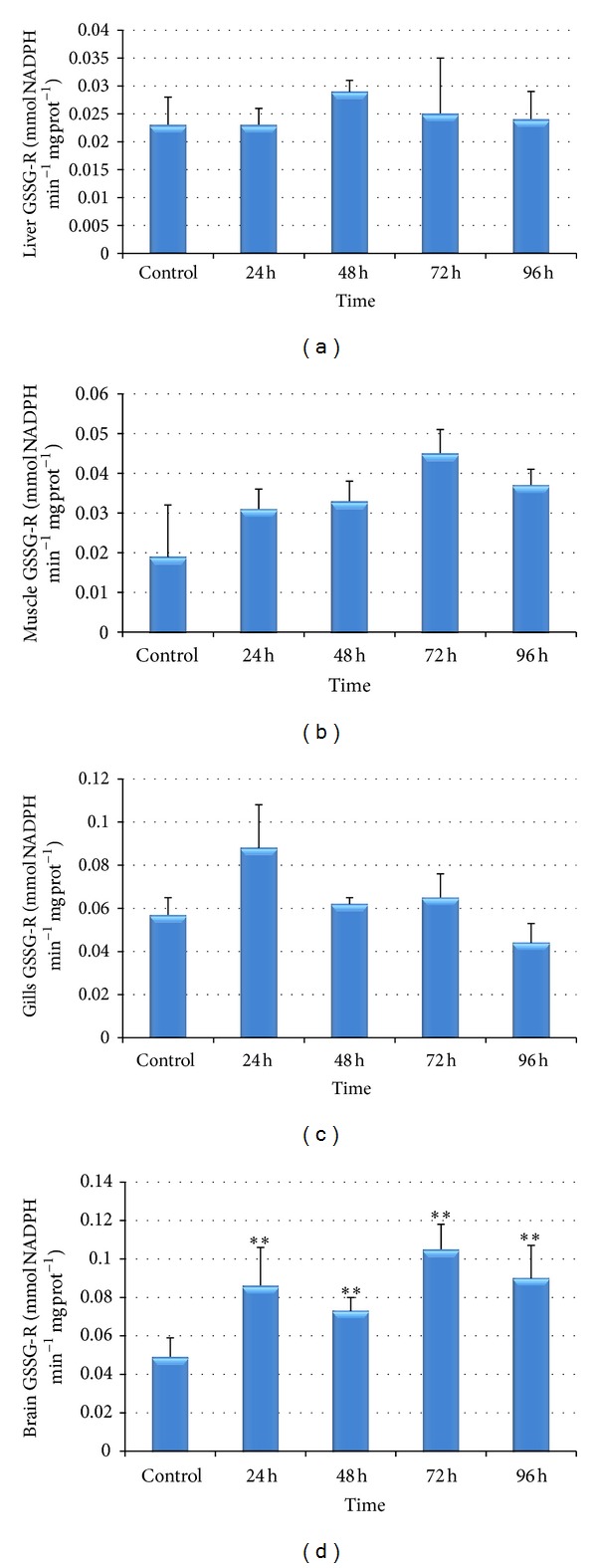
(a)–(d) Effect of hexavalent chromium on glutathione reductase (GSSG-R) activity in liver (a), muscle (b), gills (c), and brain (d) of* Labeo rohita* (values are mean ± S.D.); S.D. = standard deviation; ** = significant at (*P* < 0.05).

The effects of Cr(VI) exposure on glutathione reductase activity in fish liver, muscle, gills, and brain are presented in Figures 5(a)–5(d). An increase in activity was observed in glutathione reductase (GSSG-R) in the fishes exposed to chromium; however the increase was not significant (*P* > 0.05) except in brain.

### 3.4. Bioaccumulation

The present study was planned to investigate accumulation of heavy metal chromium with respect to short term and chronic exposure. Fishes exposed to 1/3rd of LC_50_ were kept in 5 different groups and accumulation patterns of the metal in fish body organs were investigated after 1, 2, 3, 4, and 15 days.

**Figure 6:**
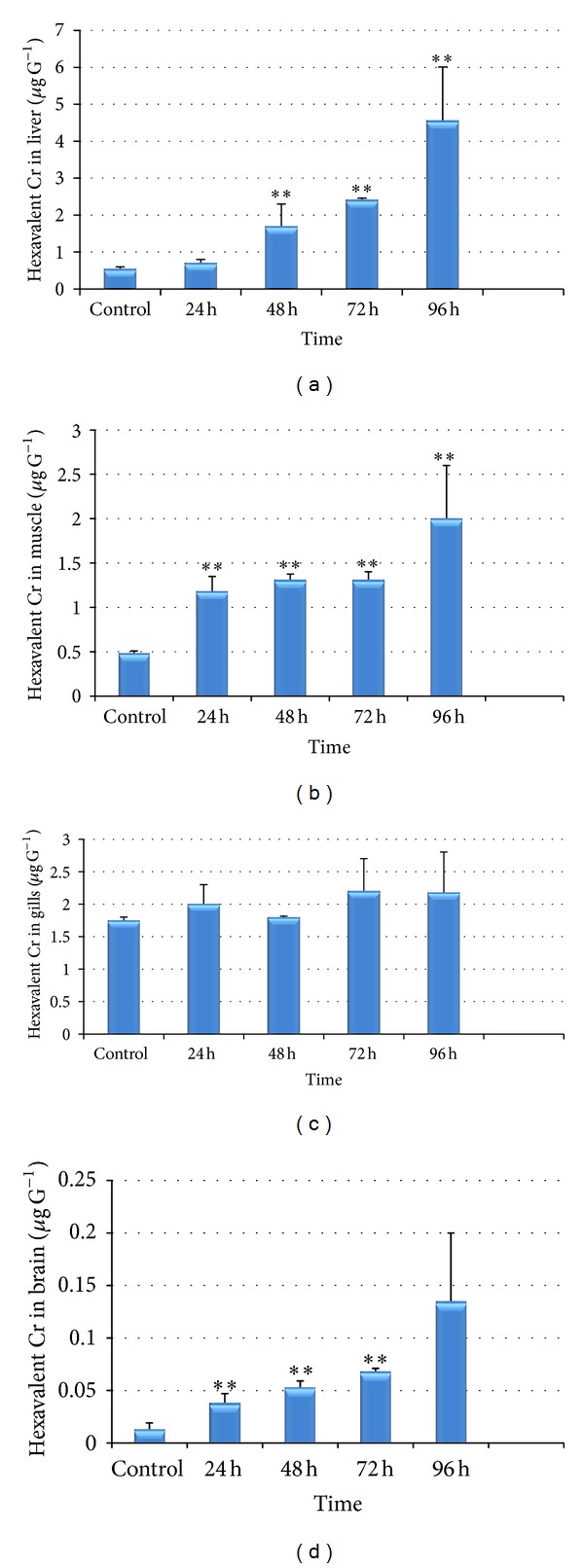
(a)–(d) Bioaccumulation of hexavalent chromium after short term exposure (24–96 hrs) in liver (a), muscle (b), gills (c), and brain (d) of* Labeo rohita* (values are mean ± S.D.); S.D. = standard deviation; ** = significant at (*P* < 0.05).

**Figure 7:**
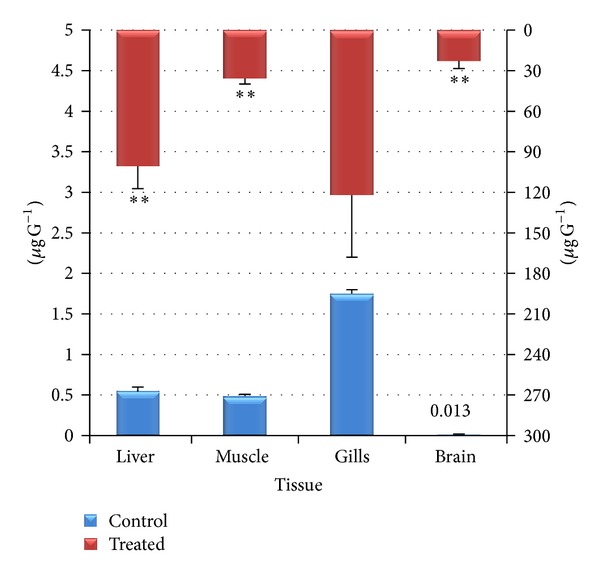
Bioaccumulation of hexavalent chromium after long term exposure (15 days) in liver, muscle, gills, and brain of* Labeo rohita* (values are mean ± S.D.); S.D. = standard deviation; ** = significant at (*P* < 0.05).

Among various body organs, the liver of the fish* Labeo rohita* sampled during short term exposure showed significantly higher concentration of chromium, followed by that of gills, muscles, and brain (Figure 6). However, it was very interesting to note that after 15 days of exposure the highest concentration was observed in gills followed by liver, muscle, and brain (Figure 7).

## 4. Discussion

### 4.1. Behavioral Response

Behavior provides a unique perspective linking the physiology and ecology of an organism and its environment [34] and allows the organism to adjust to external and internal stimuli in order to best meet the challenge of surviving in a changing environment. Chromium (VI) actively enters cells through an anion (phosphate) transport mechanism. Chromium (III), meanwhile, is not able to use this mechanism [35]. Fish mucus can reduce the oxidative state of Cr(VI) and decrease its penetration, providing fish protection against chromium pollution [36]. The copious mucus secretion observed in the present study (Table 1) is one of the selective responses of the chromium treated fishes to avoid the entry of toxicants to body. Development of locomotory responses, frequency of swimming movements, and duration of activity were significantly altered in chromium treated fishes. The results obtained in our study are supported by numerous studies showing that toxicants can disrupt startle responses [37] and the swimming performance and activity of the fishes [38–40]. The lethargic movement and loss of equilibrium support the findings in Cr(VI) treated* Channa punctatus *[41]. Normal respiration was found to be altered in the fishes treated with chromium. Respiration is a rhythmic neuromuscular sequence regulated by an endogenous biofeedback loop as well as by external environmental stimuli. Acute contaminant exposure can induce reflexive cough and gills purge responses to clear the opercular chamber of the irritant and can also increase rate and amplitude of the respiratory cycle as the fish adjusts the volume of water in the respiratory stream. As exposure continues, the respiration cycle can become irregular, largely through decreased input as well as alterations in the endogenous pacemaker. Diamond et al. [42] also found that the frequency and amplitude of bluegill opercular rhythms and cough responses were altered following exposure to different contaminants. All the above symptoms could also be due to the inhibition of acetylcholinesterase (AchE) activity leading to accumulation of acetylcholine in cholinergic synapses ensuring hyperstimulation [26].

### 4.2. Biometric Parameters (HSI and CF)

Fishes are especially susceptible to environmental variations and respond more sensitively to pollutants than mammals. The fish liver has been shown to be a very interesting model for studying the interactions between environmental factors and hepatic structure and function. Hepatosomatic index (HSI) has been frequently used as a biomarker for examining fish exposed to environmental toxicants. HSI values are generally elevated in vertebrates experiencing induction of hepatic microsomal P-450 for detoxification of the pollutants [43]. One of the functions of liver is the biotransformation and elimination of xenobiotics. Increases in the liver size are commonly seen in fish that have been exposed to xenobiotics. The increase in size is due to hyperplasia (increase in cell number), hypertrophy (increase in cell size), or both [44, 45]. The concurrent increase in HSI can indicate an increased capacity of the liver to metabolize xenobiotics in the presence of pollution.

In the present study (Figure 1) HSI increased significantly during the entire period of study in* L. rohita *due to chromium treatment indicating that the liver size increases due to metabolization of metal in the liver. This high increase of HSI shows that HSI can also be considered as a biomarker of metal toxicity.

The CF is a measure of the fattiness of the fish and this is based on the ratio between body weight and length: 100 × body weight (g)/length (cm^3^), which allows comparisons to be made between populations living under different conditions [45].

The overall decrease in CF in the present study in* L. rohita* (Figure 2) due to chromium treatment is indicative of adverse effect on health of the fish caused by the chromium.

### 4.3. Biomarkers of Oxidative Stress

The antioxidant enzymatic system protects organisms from the toxic effects of the activated oxygen species and helps to maintain cellular homeostasis by removing ROS. The use of antioxidant profiles, particularly as a function of heavy metal exposure, is of high toxicological relevance. The level of certain biomarkers of oxidative stress was evaluated in* Labeo rohita *exposed to sublethal concentration of chromium. Fish upon exposure to pollutants elicit the production of reactive oxygen species (ROS) like superoxide anion, hydrogen peroxide, and hydroxyl radical [46]. As the ROS levels increase, the biological system develops a first line defense mechanism by modulating the activities of antioxidants such as catalase (CAT), superoxide dismutase (SOD), and glutathione related enzymes [47, 48].

The biochemical profiles for catalase, GSSG, and SOD in different tissues are represented in Figures 3–5. In the present study, catalase activity increased in liver, muscle, gills, and brain of the fish exposed to sublethal concentrations of hexavalent chromium (Figures 3(a)–3(d)). Thus, the results clearly infer chromium-induced oxidative stress. H_2_O_2_ is a major component of the ROS produced intracellularly during physiological and pathological processes, in addition to being the cause of oxidative damage [49]. Catalase (H_2_O_2_ : H_2_O_2_ oxidoreductase; EC1.11.1.6), a hydrogen peroxide scavenger, catalyzes the breakdown of hydrogen peroxide to water and molecular oxygen to protect cells against the toxic effects of hydrogen peroxide [50]. The increase in catalase activity during experimental periods is probably a response to chromium induced toxic stress and serves to neutralize the impact of increased ROS generation [51]. Similar observation was made by Li et al. [52] in the brain of rainbow trout (*Oncorhynchus mykiss*) after chronic carbamazepine treatment and in the liver, gills, and kidney of* Labeo rohita* after lethal and sublethal concentrations of malathion [11].

Cr(VI) is reduced intracellularly to the reactive intermediates Cr(V) and (IV) and ultimately to the more stable Cr(III), by cellular reductants [53]. Diverse intracellular reductants have been suggested as contributors to the reduction of Cr(VI), which may either directly induce an increased production of reactive oxygen species (ROS) by catalyzing a Fenton-like redox cycling mechanism [54] or indirectly promote oxidative stress by interacting with mitochondria [55]. Reactive oxygen species can attack multiple cellular constituents including protein, nucleic acids, and lipids, leading to disruption of cellular function and integrity [56, 57].

Among antioxidant enzymes, SOD is considered as the first line of defence against oxygen toxicity, due to its inhibitory effects on oxyradical formation [58, 59]. The dismutation of the superoxide anion radical is catalyzed by SOD to water and hydrogen peroxide, which afterwards is detoxified by catalase. Therefore, a simultaneous activity induction of SOD and CAT is usually an expected response [60]. However, this relation is not always observed [61] and it is known to be species dependent [62]. In the present study, high activity values of both SOD and CAT have been observed, suggesting a “cooperative” mechanism of the two enzymatic systems. In liver, it has been noticed that the catalase activity reached its maximum level during 72 hrs, whereas in other tissues catalase achieved its maximum peak during 48 hrs (Figures 3(a)–3(d)).  Figure 4(a) clearly indicates that SOD level was maximum during 48 hrs in liver due to which ample amount of hydrogen peroxide might have generated and this could be the reason that catalase activity reached its maximum peak during 72 hrs (Figure 3(a)) in order to remove these peroxides.

One very unique and interesting finding was observed in the present study that the SOD increased from very initial period of stress (i.e., 24 hrs) and decreased or returned to normalcy during 96 hrs in all the tissues except brain, whereas CAT increased its maximum level during 48 hrs to 72 hrs and did not return to normalcy even in 96 hrs (except muscle). Therefore, it may be concluded based on the results that SOD takes the lead in order to detoxify the oxyradicals followed by corresponding increase in CAT activity and both are time-dependent.

We also observed that SOD and CAT activity in brain was strongly induced by Cr(VI) even during 96 hrs of treatment, which could be due to the flux of superoxide radicals, resulting in increased H_2_O_2_ in the cells [63, 64] leading to prolonged induction of SOD and CAT. The significantly induced CAT and SOD also indicates the stronger antioxidant defense capability of liver [59] and brain.

In free radical scavenging and other cellular metabolism, reduced glutathione and GSH-related enzymes play a major role [65–67]. They may also be involved in Cr(VI) detoxification, and in many cases glutathione may be active [13]. During the reduction of Cr(VI), GSH may be oxidized to GSSG, which would normally be recycled back to GSH by the activity of glutathione reductase using NADPH as the electron donor [68].

In our study, we did not find significant elevations in glutathione reductase activity in liver, muscle, and gills tissues. Experiment conducted on Zebra fish as a function of norfloxacin by Bartoskova et al. [69] has also not found any significant difference in glutathione reductase activity. However, in brain, the activity was found to be significantly increased in all the period of treatment. Li et al. [70] have also found significant induction in glutathione reductase (GR) activities in fish brain* in vivo* evaluating the toxicity of environmental concentrations of waterborne chromium (VI) to a model teleost,* Oncorhynchus mykiss*. Significantly elevated GR activities in brain might suggest a critical role for this enzyme in brain cell protection against the deleterious effects of chromium.

Thus, the increase in catalase, SOD, and GR activity observed in the current study suggests that Cr(VI) is capable of inducing oxidative stress to the fish* Labeo rohita*. Similar findings reported earlier are in agreement with these findings [47, 71]. Cellular biomarkers can reveal the early onset of biological alterations due to chemical pollutants, with a prognostic or diagnostic value toward long-term toxicological or ecological effects [72]. Therefore, CAT, SOD, and GR activity could be used as biomarkers of metal pollution and be implemented in biomonitoring program in areas potentially polluted with metals to assess the health of the ecosystem.

### 4.4. Bioaccumulation

Knowledge of concentrations of heavy metal in different tissues of fish is important with respect to nature of management and human consumption of fish. Metal accumulation in the tissues of fish varies according to the rates of uptake, storage, and elimination. This means that metals which have high uptake and low elimination rates in the tissues of fish are expected to be accumulated to higher levels.

Amongst all the tissues studied (liver, muscle, gills, and brain), the highest concentration of chromium (4.56 ± 1.45 *μ*gg^−1^) was observed in the liver of the fish* Labeo rohita* (*P* < 0.05) after 96 hrs period of exposure which was followed by gills (2.18 ± 0.62 *μ*gg^−1^); muscle (2.0 ± 0.60 *μ*gg^−1^); and brain (0.135 ± 0.065 *μ*gg^−1^) (Figures 6(a)–6(d)). After 15 days of exposure the chromium accumulation followed the following trend: gills (121.8 ± 46.22 *μ*gg^−1^); liver (100.80 ± 16.6 *μ*gg^−1^); muscle (35.65 ± 4.25 *μ*gg^−1^); and brain (22.75 ± 5.60 *μ*gg^−1^) (Figure 7). The Cr bioaccumulation pattern in the selected tissues of the fish showed that highest concentrations of Cr were mostly recorded in the liver and gills and minimum concentration was recorded in muscle and brain. In the control fish however, small amount of chromium was present which may be acquired from the sediment through food chain and via gills and intestine [73].

The liver exhibited highest tendency to accumulate chromium metal, whereas the most consumed part of the fish, that is, muscle, showed least tendency. Liver and gills of fish species, namely,* Sparus aurata*,* Dicentrarchus labrax*,* Mugil cephalus*, and* Scomberomorus cavalla*, have been reported to accumulate highest levels of cadmium, lead, copper, zinc, and iron [74, 75]. A study conducted on* Leuciscus cephalus* and* Lepomis gibbosus* [76] reported maximum accumulations of cadmium, cobalt, and copper in the liver and gills, while these accumulations were least in the fish muscle. The higher levels of trace elements such as lead and chromium in liver relative to other tissues may be attributed to the affinity or strong coordination of metallothionein protein with these elements [77]. Low levels of chromium in fish muscles and brain appear to be due to low levels of binding proteins. A study conducted on* Cyprinus carpio*,* Barbus capito*, and* Chondrostoma regium* caught at 5 stations on the Seyhan river system has also confirmed the maximum accumulation of cadmium and chromium in the liver and gills than muscles [78]. Thus, heavy metals when present in the water source can enter the food chain and accumulate in the fish that are often on the top of food chain and have the tendency to accumulate heavy metals [79]. Therefore, bioaccumulation of metals in fish can be considered as an index of metal pollution in the aquatic bodies [80, 81] that could be a useful tool to study the biological role of metals present at higher concentrations in fish [74].

## 5. Conclusion

The results of the present study clearly indicate that heavy metal chromium causes oxidative stress in fishes. Increased CAT, SOD, and GR activities in all organs might suggest the crucial role of these enzymes in cell protection against the deleterious effects of chromium and development of adaptive response to chromium toxicity. The current results also contribute to improving our knowledge about possible development of oxidative stress induced by exposure to chromium metal in aquatic organisms and indicate a possible role for antioxidant systems in the prevention of induced damage. Bioaccumulation of chromium varied between the different tissues and the liver, being the storage and detoxification organ, accumulated highest level during short term exposure followed by gills. The gills had the highest metal concentrations during long term exposure, due to their intimate contact with the environment and their importance as an effecter of ionic and osmotic regulation. Muscle accumulated much less chromium. Thus, it is proposed to include these parameters in biomonitoring program in areas potentially polluted with metals to assess the health of the ecosystem.

## Figures and Tables

**Table 1:** Behavioral changes in the fish *Labeo rohita* as a function of hexavalent chromium.

S.N.	Behavioral changes
1	Surfacing and darting movement
2	Copious mucus secretion
3	Aggregation of fishes near aerator
4	Lethargic movement within 20 minutes after exposure
5	Increase in opercular movement in order to breathe faster
6	Irregular and burst swimming-sudden rapid and forward movements
7	Nip and nudge movement-biting and moving towards another fish
8	Fin flickering-contraction and extension of dorsal fin
